# Placental Morphology Is Associated with Maternal Depressive Symptoms during Pregnancy and Toddler Psychiatric Problems

**DOI:** 10.1038/s41598-017-19133-9

**Published:** 2018-01-15

**Authors:** Marius Lahti-Pulkkinen, Melissa Jane Cudmore, Eva Haeussner, Christoph Schmitz, Anu-Katriina Pesonen, Esa Hämäläinen, Pia M. Villa, Susanna Mehtälä, Eero Kajantie, Hannele Laivuori, Rebecca M. Reynolds, Hans-Georg Frank, Katri Räikkönen

**Affiliations:** 1 Department of Psychology and Logopedics, University of Helsinki, Helsinki, Finland; 2 British Heart Foundation Centre for Cardiovascular Science, Queen’s Medical Research Institute, University of Edinburgh, Edinburgh, United Kingdom; 3 Chronic Disease Prevention Unit, National Institute for Health and Welfare, Helsinki, Finland; 4 Department of Anatomy II, LMU Munich, Munich, Germany; 5 HUSLAB and Department of Clinical Chemistry, Helsinki University Central Hospital, Helsinki, Finland; 6 Obstetrics and Gynaecology, University of Helsinki and Helsinki University Hospital, Helsinki, Finland; 7 Medical and Clinical Genetics, University of Helsinki and Helsinki University Hospital, Helsinki, Finland; 8 PEDEGO Research Unit, MRC Oulu, Oulu University Hospital and University of Oulu, Oulu, Finland; 9 Children’s Hospital, Helsinki University Hospital and University of Helsinki, Helsinki, Finland; 10 Institute for Molecular Medicine Finland, HiLIFE Unit, University of Helsinki, Helsinki, Finland; 11 Faculty of Medicine and Life Sciences, University of Tampere, Tampere, Finland; 12 Department of Obstetrics and Gynecology, Tampere University Hospital, Tampere, Finland

## Abstract

Maternal depressive symptoms during pregnancy predict increased psychiatric problems in children. The underlying biological mechanisms remain unclear. Hence, we examined whether alterations in the morphology of 88 term placentas were associated with maternal depressive symptoms during pregnancy and psychiatric problems in 1.9–3.1-years old (Mean = 2.1 years) toddlers. Maternal depressive symptoms were rated biweekly during pregnancy with the Center of Epidemiological Studies Depression Scale (n = 86). Toddler psychiatric problems were mother-rated with the Child Behavior Checklist (n = 60). We found that higher maternal depressive symptoms throughout pregnancy [B = −0.24 Standard Deviation (SD) units: 95% Confidence Interval (CI) = −0.46; −0.03: P = 0.03; Mean difference = −0.66 SDs; 95% CI = −0.08; −1.23: P = 0.03; between those with and without clinically relevant depressive symptoms] were associated with lower variability in the placental villous barrier thickness of γ-smooth muscle actin-negative villi. This placental morphological change predicted higher total (B = −0.34 SDs: 95% CI = −0.60; −0.07: P = 0.01) and internalizing (B = −0.32 SDs: 95% CI = −0.56; −0.08: P = 0.01) psychiatric problems in toddlers. To conclude, our findings suggest that both maternal depressive symptoms during pregnancy and toddler psychiatric problems may be associated with lower variability in the villous membrane thickness of peripheral villi in term placentas. This lower heterogeneity may compromise materno-fetal exchange, suggesting a possible role for altered placental morphology in the fetal programming of mental disorders.

## Introduction

Maternal clinically relevant depressive symptoms complicate up to 10–20% of pregnancies^1–5^. Maternal depressive symptoms not only disrupt the health of the pregnant woman^4,6^ but also have adverse consequences on offspring mental health^2,3,7–9^. Several studies have shown that maternal depressive symptoms during pregnancy predict increased psychiatric problems in offspring, and these effects are not explained by maternal depressive symptoms after pregnancy^2,3,7–9^. For example, we showed among 2296 Prediction and Prevention of Preeclampsia and Intrauterine Growth Restriction (PREDO)-study participants that maternal depressive symptoms during pregnancy predict increased psychiatric problems in children, independently of maternal depressive symptoms after pregnancy^3^. However, the biological mechanisms underlying these associations remain largely unknown.

Emerging evidence suggests that maternal depressive symptoms during pregnancy may alter maternal physiological homeostasis above and beyond alterations induced by the pregnancy itself, leading to changes in hypothalamic-pituitary-adrenocortical axis, inflammatory and autonomic nervous system functioning, and in oxidative stress and nutrition levels^2,6,9–14^. These depression-related physiological changes may alter the normal placental adaptation and remodelling which occurs to ensure that the requirements for oxygen, hormones, nutrients and waste removal of the growing fetus are met throughout gestation^15^.

Aberrations in placental development and maturation that adversely affect the capacity of the placenta to provide optimal materno-fetal exchange can influence fetal health and program the fetus for disease development later in life^16,17^. A mature placenta comprises an extensive villous tree containing a network of fetal capillaries that bathes in a pool of maternal blood^18^. The villous tree consists of large structural stem villi that arborize to form intermediate and terminal villi, with a γ-smooth muscle actin(SMA)-positive perivascular layer of myofibroblast-like cells that aborize in the (then myofibroblast-free and SMA-negative) peripheral part of the villous tree^19^. This peripheral part of the villous tree facilitates most materno-fetal exchange^20^. The whole villous tree is covered by a principally bi-layered epithelium with an at-term largely incomplete basal layer of cytotrophoblasts and an apical layer, called the syncytiotrophoblast. The layers from the apical aspect of the syncytiotrophoblast to the apical (luminal) surface of the fetal capillary endothelium constitute the placental villous membrane^21^, the villous materno-fetal barrier^15^. As pregnancy progresses and fetal demand for gaseous and nutrient exchange increases, the villous membrane thickness becomes irregular^22^. Some areas become extremely thin as locally dilated segments of fetal capillaries protrude into the trophoblast layer and some areas remain thicker, where syncytial organelles and nuclei accumulate outwith these thin barrier areas^18,23^. The areas of extremely thin villous membrane, as small as 1–2 microns, are known as vasculo-syncytial membranes (VSM) and are the primary materno-fetal exchange sites^24^. Thus, a healthy late gestation placenta has high variability in villous membrane thickness measures and hence diffusion distance evidenced by larger standard deviations (SD:s)^25^. Increased VSM thickness and a resulting decrease in villous membrane thickness variability has been shown in placentas from pregnancies complicated by preeclampsia^26^ and gestational diabetes^27^.

Previous studies have shown that maternal psychological distress during pregnancy may be associated with altered placental weight and fetoplacental circulation^28,29^, and alterations in placental weight and/or surface area may predict psychiatric disturbance in childhood and adolescence^30^, and certain personality disorders^31^ and traits^32^ in adulthood. Yet, no previous studies have tested associations between maternal depressive symptoms or child psychiatric problems and placental morphology. In this hypothesis-generating study, we tested whether maternal depressive symptoms during pregnancy are associated with alterations in the morphology of term placentas, and if term placental morphology predicts child psychiatric problems in toddlerhood.

## Results

### Participant Characteristics and Potential Confounders

Table 1 shows the sample characteristics. In our study sample, maternal depressive symptoms showed high inter-individual stability across pregnancy trimesters (r’s from =0.63 to =0.84, P < 0.001). Toddler internalizing, externalizing and total psychiatric problems also had high inter-correlations (r’s from =0.63 to =0.88, P < 0.001). Supplementary Table S1 shows the intercorrelations between the different placental morphology indicators.

**Table 1:** Characteristics of the Study Sample.

Maternal Characteristics	Data Available(N)	Mean(SD^a^)/N(%)
Age at Delivery (years)	88	31.5 (4.9)
Education, Tertiary	88	57 (64.8%)
Pre-Pregnancy Body Mass Index (kg/m^2^)	88	24.0 (4.2)
Any Diabetic or Hypertensive Disorder in Pregnancy (type 1 or gestational diabetes, chronic or gestational hypertension, pre-eclampsia), Yes	88	15 (17.0%)
History of Physician-Diagnosed Mental Disorders Before Pregnancy, Yes	80	13 (16.3%)
Depressive Symptoms		
Trimester-Weighted Mean Score of Center for Epidemiological Studies	86	10.3 (6.0)
Depression Scale Depressive Symptoms during Pregnancy		
Trimester-Weighted Mean Score of Center for Epidemiological Studies	86	14 (16.3%)
Depression Scale Depressive Symptoms during Pregnancy ≥ 16		
1^st^ Pregnancy Trimester Center for Epidemiological Studies Depression	81	10.2 (6.7)
Scale Depressive Symptom Score		
Mean of 2^nd^ Pregnancy Trimester Center for Epidemiological Studies	86	10.4 (6.5)
Depression Scale Depressive Symptom Scores		
Mean of 3^rd^ Pregnancy Trimester Center for Epidemiological Studies	86	10.3 (6.6)
Depression Scale Depressive Symptom Scores		
Number of Pregnancy Trimesters with Center for Epidemiological Studies	81	
0		53 (65.4%)
1		16 (19.8%)
2–3		12 (14.8%)
Beck Depression Inventory-II Score Concurrently to Rating the Child	60	5.9 (5.5)
Placental Morphology		
Volume of SMA^b^-Positive Villi per Placenta (ml)	88	95.1 (50.9)
Volume of Capillaries per SMA-Positive Villi per Placenta (ml)	88	56.0 (35.6)
Volume of SMA^b^-Negative Villi per Placenta (ml)	88	182.7 (63.9)
Volume of Capillaries per SMA^b^-Negative Villi per Placenta (ml)	88	157.5 (51.8)
Ratio of the Distribution of SMA^b^-Positive Villi to SMA-Negative Villi (%)	88	63.7 (56.8)
Villous Barrier Thickness of SMA^b^-Positive Villi (µm)	88	7.5 (1.7)
Standard Deviation of Villous Barrier Thickness of SMA^b^-Positive Villi (Variability in Thickness) (µm)	88	6.3 (2.3)
Villous Barrier Thickness of SMA^b^-Negative Villi (µm)	88	11.9 (4.6)
Standard Deviation of Villous Barrier Thickness of SMA^b^-Negative Villi (µm)	88	10.2 (5.5)
Placental Weight (grams)	88	589.7 (107.4)
Toddler Characteristics		
Gestation Length (weeks)	88	40.3 (1.0)
Sex	88	
Boys		41 (46.6%)
Girls		47 (53.4%)
Age at Follow-up (months)	60	25.5 (2.9)
Child Behavior Checklist/1½-5 Psychiatric Problems		
Total Problems	60	46.8 (9.2)
Internalizing Problems	60	45.4 (8.5)
Externalizing Problems	60	48.6 (9.4)

^a^SMA = γ-smooth muscle actin. ^b^SD = standard deviation.

Maternal diabetic and hypertensive disorders in pregnancy were associated with smaller capillary volumes per SMA-positive villi per placenta (Mean Difference (MD) = 0.56: 95% Confidence Interval (CI) = 0.00;1.12: P = 0.05). Girls had larger capillary volumes per SMA-negative villi per placenta than boys (MD = 0.41: 95%CI = 0.01;0.81: P = 0.05). Gestation length was positively associated with volumes of SMA-negative villi (r = 0.24; P = 0.02) and capillaries per SMA-negative villi (r = 0.27; P = 0.01) per placenta. Maternal age, pre-pregnancy body mass index (BMI), education, or history of mental disorders before pregnancy were not associated with placental morphology (P-values ≥ 0.10).

### Maternal Depressive Symptoms during Pregnancy and Placental Morphology

**Figure 1:**
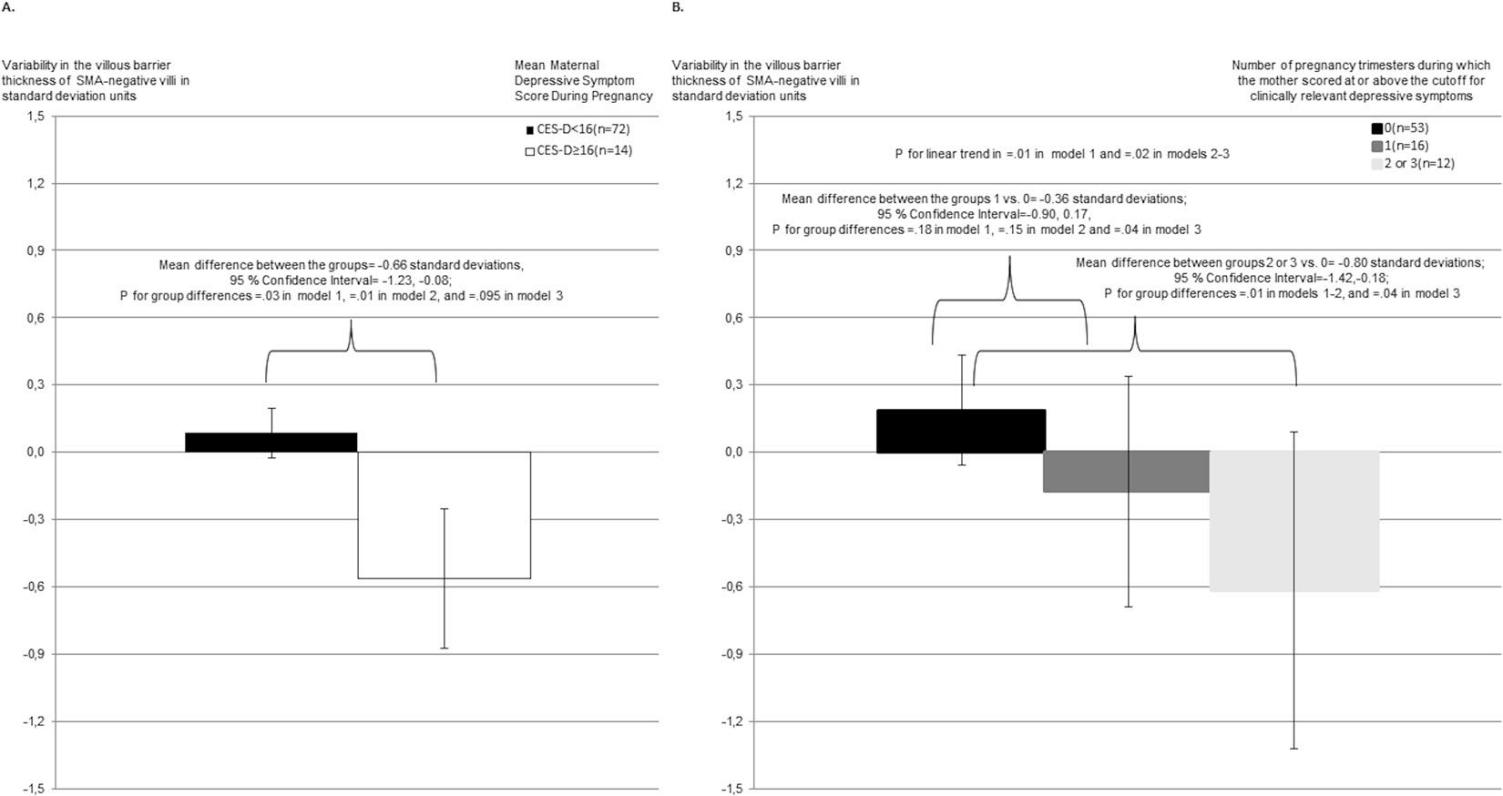
(**A**) Maternal clinically significant antenatal depressive symptoms and placental SMA-negative villi villous barrier thickness variability. The figure shows the unadjusted mean values and 95% Confidence Intervals of the standard deviation of placental SMA-negative villi villous barrier thickness (in standard deviation units) for mothers with trimester-weighted mean antenatal depressive symptoms below or above the clinical cutoff of ≥16 points. P-values refer to group difference significances from unadjusted linear regression models (model 1), models adjusted for maternal age, education, pre-pregnancy BMI, hypertensive and diabetic disorders in pregnancy and gestation length (model 2), and models adjusted also for maternal history of physician-diagnosed mental disorders before pregnancy (model 3). (**B**) Maternal accumulative depressive symptoms during pregnancy and SMA-negative villi villous barrier thickness variability. The figure shows the unadjusted mean values and 95% Confidence Intervals of the standard deviation of SMA-negative villi villous barrier thickness in standard deviation units, according to the number of pregnancy trimesters [0, 1, 2–3] maternal depressive symptom scores during pregnancy were above the clinical cutoff of ≥16, when calculated from the value at the first trimester and mean values across second and third trimesters. P-values refer to the significances of group differences and linear trends from unadjusted linear regression models (model 1), models adjusted for maternal age, education, pre-pregnancy BMI, hypertensive and diabetic disorders in pregnancy and gestation length (model 2), and models adjusted also for maternal history of physician-diagnosed mental disorders before pregnancy (model 3).

Higher maternal depressive symptoms throughout pregnancy (Table 2) and during each pregnancy trimester (Table 3) were associated with a significantly smaller SD of the villous barrier thickness of SMA-negative villi (indicating less villous barrier thickness variability) in unadjusted and/or adjusted regression analyses. Figure 1 shows that similar associations with a smaller SD of SMA-negative villi villous barrier thickness were found when CES-D depressive symptoms throughout pregnancy were above the clinical cutoff score (Panel A), and when these symptoms were more frequently above the cutoff during pregnancy trimesters (Panel B). The associations with this placental morphology indicator were most consistent across analytic models for maternal depressive symptoms during the first pregnancy trimester (Table 3).

**Table 2:** Maternal Depressive Symptoms across Pregnancy and Placental Morphology. Unstandardized regression coefficients (B) and their 95% Confidence Intervals (CI) indicating standard deviation unit increase in placental morphology criteria per one standard deviation increase in maternal depressive symptoms across pregnancy from linear regression models where maternal depressive symptoms during pregnancy were used as predictors of placental morphology criteria.

Placental Morphology Criteria	Maternal Depressive Symptoms during Pregnancy					
	Model 1^a^		Model 2^b^		Model 3^c^	
	B(95% CI)	p	B(95% CI)	p	B(95% CI)	p
Volume of SMA-positive villi per placenta	0.06(−0.15;0.28)	0.56	0.11(−0.11;0.33)	0.34	0.11(−0.13;0.34)	0.36
Volume of capillaries per SMA-positive villi per placenta	0.05(−0.17;0.27)	0.65	0.09(−0.13;0.31)	0.42	0.10(−0.14;0.34)	0.41
Volume of SMA-negative villi per placenta	−0.09(−0.33;0.14)	0.43	−0.08(−0.31;0.14)	0.47	−0.06(−0.30;0.18)	0.64
Volume of capillaries per SMA-negative villi per placenta	−0.13(−0.35;0.09)	0.26	−0.12(−0.34;0.10)	0.28	−0.10(−0.33;0.13)	0.40
Ratio of the distribution of stem villi to SMA-negative villi	0.13(−0.09;0.34)	0.26	0.16(−0.06;0.38)	0.15	0.16(−0.07;0.40)	0.18
Villous barrier thickness of SMA-positive villi	−0.10(−0.32;0.12)	0.36	−0.12(−0.35;0.11)	0.31	−0.06(−0.30;0.18)	0.60
Standard deviation of villous barrier thickness of SMA-positive villi (variability in thickness)	−0.10(−0.31;0.13)	0.42	−0.08(−0.31;0.15)	0.49	−0.07(−0.32;0.18)	0.57
Villous barrier thickness of SMA-negative villi	−0.17(−0.38;0.04)	0.12	−0.21(−0.43;0.01)	0.06	−0.14(−0.37;0.09)	0.22
Standard deviation of villous barrier thickness of SMA-negative villi (variability in thickness)	−0.24(−0.46;−0.03)	0.03	−0.27(−0.50;−0.05)	0.02	−0.20(−0.43;0.04)	0.096

SMA = γ-smooth muscle actin.

^a^Model 1 refers to unadjusted linear regression models.

^b^Model 2 refers to linear regression models adjusted for gestation length, maternal age at delivery, education, pre-pregnancy body mass index and diabetic and hypertensive disorders in pregnancy.

^c^Model 3 refers to linear regression models adjusted for model 2 covariates and maternal history of mental disorders before pregnancy.

**Table 3:** Maternal Trimester-Specific Prenatal Depressive Symptoms and Placental Morphology.

Depressive Symptoms during	1^st^ Pregnancy Trimester (n = 81)		2^nd^ Pregnancy Trimester (n = 86)		3^rd^ Pregnancy Trimester (n = 86)	
Placental Criteria	B(95% CI)	p	B(95% CI)	p	B(95% CI)	p
Volume of SMA-positive villi per placenta						
Model 1	0.01(−0.22; 0.23)	0.97	0.05(−0.16; 0.26)	0.61	0.12(−0.09; 0.33)	0.27
Model 2	0.06(−0.18; 0.31)	0.61	0.10(−0.11; 0.32)	0.33	0.14(−0.07; 0.36)	0.19
Model 3	0.07(−0.18; 0.32)	0.59	0.10 (−0.12; 0.32)	0.37	0.15 (−0.08; 0.38)	0.21
Volume of capillaries per SMA-positive villi per placenta						
Model 1	0.00(−0.23; 0.23)	0.99	0.04(−0.17; 0.25)	0.68	0.11(−0.11; 0.32)	0.33
Model 2	0.07(−0.17; 0.32)	0.55	0.09(−0.13; 0.30)	0.43	0.12(−0.10; 0.34)	0.27
Model 3	0.09(−0.17; 0.34)	0.49	0.09(−0.14; 0.32)	0.43	0.14(−0.10; 0.37)	0.25
Volume of SMA-negative villi per placenta						
Model 1	0.05(−0.19; 0.29)	0.70	−0.07(−0.29; 0.14)	0.48	−0.16(−0.37; 0.06)	0.15
Model 2	−0.01(−0.26; 0.25)	0.97	−0.05(−0.27; 0.17)	0.66	−0.13(−0.35; 0.09)	0.23
Model 3	0.01(−0.26; 0.28)	0.94	0.02(−0.25; 0.21)	0.86	−0.11(−0.35; 0.13)	0.36
Volume of capillaries per SMA-negative villi per placenta						
Model 1	−0.02(−0.22; 0.26)	0.88	−0.12(−0.33; 0.09)	0.27	−0.18(−0.40; 0.03)	0.09
Model 2	−0.07(−0.32; 0.18)	0.59	−0.08(−0.29; 0.13)	0.46	−0.15(−0.36; 0.07)	0.17
Model 3	−0.06(−0.32; 0.20)	0.66	−0.06(−0.28; 0.17)	0.61	−0.13(−0.37; 0.10)	0.26
Ratio of the distribution of stem villi to SMA-negative villi						
Model 1	0.01(−0.23; 0.25)	0.92	0.11(−0.10; 0.32)	0.30	0.20(−0.01; 0.41)	0.07
Model 2	0.09(−0.16; 0.35)	0.47	0.14(−0.07; 0.36)	0.19	0.21(−0.01; 0.42)	0.06
Model 3	0.09(−0.17; 0.36)	0.48	0.14(−0.09; 0.36)	0.23	0.22(−0.02; 0.45)	0.07
Villous barrier thickness of SMA-positive villi						
Model 1	−0.09(−0.33; 0.15)	0.45	−0.06(−0.27; 0.15)	0.58	−0.14(−0.35; 0.08)	0.20
Model 2	−0.13(−0.39; 0.13)	0.33	−0.07(−0.29; 0.15)	0.52	−0.15(−0.38; 0.07)	0.18
Model 3	−0.08(−0.34; 0.18)	0.55	−0.03(−0.25; 0.20)	0.82	−0.10(−0.34; 0.14)	0.40
Standard deviation of villous barrier thickness of SMA-positive villi (variability in thickness)						
Model 1	−0.06(−0.30; 0.19)	0.65	−0.07(−0.28; 0.14)	0.49	−0.10(−0.32; 0.11)	0.34
Model 2	−0.06(−0.32; 0.21)	0.67	−0.07(−0.29; 0.16)	0.56	−0.10(−0.33; 0.13)	0.38
Model 3	−0.04(−0.32; 0.23)	0.76	−0.06(−0.29; 0.18)	0.63	−0.10(−0.34; 0.15)	0.45
Villous barrier thickness of SMA-negative villi						
Model 1	−0.16(−0.40; 0.08)	0.18	−0.16(−0.36; 0.04)	0.12	−0.17(−0.38; 0.04)	0.11
Model 2	−0.25(−0.50; 0.00)	0.052	−0.19(−0.40; 0.03)	0.08	−0.19(−0.40; 0.02)	0.08
Model 3	−0.19(−0.44; 0.06)	0.14	−0.12(−0.34; 0.09)	0.26	−0.11(−0.34; 0.12)	0.35
Standard deviation of villous barrier thickness of SMA-negative villi (variability in thickness)						
Model 1	−0.24(−0.48; −0.01)	0.04	−0.20(−0.40; 0.01)	0.06	−0.22(−0.43; −0.01)	0.04
Model 2	−0.33(−0.58; −0.08)	0.01	−0.23(−0.44; −0.01)	0.05	−0.23(−0.45; −0.01)	0.04
Model 3	−0.27(−0.53; −0.02)	0.04	−0.15(−0.37; 0.07)	0.18	−0.14(−0.37; 0.10)	0.24

SMA = γ-smooth muscle actin.

Unstandardized regression coefficients (B) and their 95% Confidence Intervals (CI) from linear regression models, indicating standard deviation unit increase in placental morphology criteria per one standard deviation increase in mean maternal depressive symptoms during each pregnancy trimester.

Regression Model 1 is unadjusted. Model 2 is adjusted for maternal age and education level, pre-pregnancy body mass index, hypertensive and diabetic disorders in pregnancy and gestation length. Model 3 is adjusted for Model 2 covariates and maternal history of mental disorders before pregnancy.

### Placental Morphology and Child Psychiatric Problems

A smaller SD of the villous barrier thickness of SMA-negative villi predicted significantly higher toddler total and internalising psychiatric problems in unadjusted and adjusted regression models (Table 4). A smaller SD of SMA-negative villi villous barrier thickness also predicted higher toddler sleep, affective, anxiety, anxious/depressed, emotionally reactive, pervasive developmental and oppositional defiant problems in unadjusted and/or adjusted regression models (Supplementary Table S2).

**Table 4:** Placental Morphology and Toddler Psychiatric Problems. Unstandardized regression coefficients (B) and their 95% Confidence Intervals (CI) indicating standard deviation unit increase in toddler psychiatric problems per standard deviation increase in placental morphology criteria from linear regression models where placental morphology criteria were used to predict toddler psychiatric problems.

Toddler Psychiatric Problem Scale	Total Problems		Internalizing Problems		Externalizing Problems	
Placental Morphology Structure	B(95% CI)	p	B(95% CI)	p	B(95% CI)	p
Volume of SMA-positive villi per placenta						
Model 1	0.02(−0.23; 0.27)	0.88	−0.03(−0.27; 0.19)	0.77	−0.05(−0.31; 0.21)	0.70
Model 2	−0.00(−0.27; 0.27)	0.98	−0.05(−0.29; 0.19)	0.69	−0.08(−0.36; 0.20)	0.58
Model 3	−0.04(−0.20; 0.27)	0.76	−0.02(−0.23; 0.20)	0.88	−0.04(−0.29; 0.22)	0.78
Volume of capillaries per SMA-positive villi per placenta						
Model 1	0.02(−0.23; 0.27)	0.90	−0.03(−0.25; 0.20)	0.81	−0.05(−0.31; 0.21)	0.68
Model 2	−0.02(−0.29; 0.25)	0.87	−0.07(−0.30; 0.17)	0.59	−0.08(−0.36; 0.20)	0.58
Model 3	−0.01(−0.25; 0.23)	0.92	−0.05(−0.27; 0.16)	0.63	−0.08(−0.33; 0.17)	0.54
Volume of SMA-negative villi per placenta						
Model 1	−0.05(−0.32; 0.23)	0.72	0.04(−0.21; 0.29)	0.75	−0.11(−0.39; 0.18)	0.46
Model 2	−0.12(−0.44; 0.19)	0.44	−0.02(−0.30; 0.26)	0.88	−0.20(−0.52; 0.13)	0.22
Model 3	0.05(−0.22; 0.32)	0.71	0.13(−0.11; 0.37)	0.28	−0.03(−0.31; 0.26)	0.84
Volume of capillaries per SMA-negative villi per placenta						
Model 1	−0.09(−0.35; 0.18)	0.51	−0.00(−0.24; 0.24)	0.99	−0.12(−0.40; 0.15)	0.36
Model 2	−0.20(−0.51; 0.11)	0.21	−0.09(−0.37; 0.19)	0.51	−0.25(−0.57; 0.07)	0.13
Model 3	0.00(−0.26; 0.25)	0.98	0.08(−0.15; 0.31)	0.49	−0.06(−0.33; 0.21)	0.67
Ratio of the distribution of SMA-positive villi to SMA-negative villi						
Model 1	0.02(−0.23; 0.27)	0.88	−0.07(−0.30; 0.16)	0.54	0.00(−0.26; 0.26)	0.99
Model 2	0.03(−0.25; 0.31)	0.82	−0.06(−0.30; 0.19)	0.64	0.01 (−0.27; 0.30)	0.92
Model 3	0.01(−0.23; 0.25)	0.92	−0.08(−0.29; 0.14)	0.48	−0.00(−0.26; 0.25)	0.97
Villous barrier thickness of SMA-positive villi						
Model 1	−0.08(−0.36; 0.21)	0.60	−0.12(−0.37; 0.14)	0.36	0.02(−0.27; 0.31)	0.89
Model 2	−0.11(−0.44; 0.21)	0.48	−0.19(−0.47; 0.09)	0.18	0.01(−0.33; 0.35)	0.95
Model 3	0.02(−0.26; 0.29)	0.91	−0.04(−0.29; 0.21)	0.75	0.10(−0.19; 0.39)	0.49
Standard deviation of villous barrier thickness of SMA positive villi (variability in thickness)						
Model 1	−0.02(−0.28; 0.23)	0.86	0.02(−0.21; 0.25)	0.87	−0.05(−0.31; 0.21)	0.70
Model 2	−0.05(−0.32; 0.22)	0.71	−0.01(−0.25; 0.23)	0.93	−0.07(−0.35; 0.21)	0.62
Model 3	0.06(−0.19; 0.31)	0.63	0.09(−0.13; 0.31)	0.40	0.02(−0.24; 0.27)	0.91
Villous barrier thickness of SMA-negative villi						
Model 1	−0.14(−0.42; 0.13)	0.31	−0.18(−0.42; 0.07)	0.16	−0.00(−0.29; 0.28)	0.98
Model 2	−0.16(−0.45; 0.12)	0.26	−0.20(−0.45; 0.05)	0.12	−0.01(−0.31; 0.29)	0.93
Model 3	−0.08(−0.34; 0.19)	0.56	−0.12(−0.36; 0.12)	0.31	0.05(−0.23; 0.33)	0.71
Standard deviation of villous barrier thickness of SMA-negative villi (variability in thickness)						
Model 1	−0.34(−0.60; −0.07)	0.01	−0.32(−0.56; −0.08)	0.01	−0.20(−0.48; 0.09)	0.17
Model 2	−0.35(−0.63; −0.07)	0.01	−0.34(−0.59; −0.10)	0.01	−0.21(−0.51; 0.09)	0.16
Model 3	−0.29(−0.54; −0.03)	0.03	−0.28(−0.51; −0.05)	0.02	−0.15(−0.43; 0.13)	0.28

SMA = γ-smooth muscle actin.

Model 1 is unadjusted. Model 2 is adjusted for toddler’s age and sex, gestation length, maternal age at childbirth, education, pre-pregnancy BMI and maternal diabetic and hypertensive disorders in pregnancy. Model 3 is adjusted for maternal depressive symptoms concurrently to assessing her child’s psychiatric problems.

## Discussion

This explorative, hypothesis-generating study examined whether maternal depressive symptoms during pregnancy and subsequent toddler psychiatric problems were associated with morphology alterations of term placentas. We found that both maternal depressive symptoms across pregnancy and toddler total and internalizing psychiatric problems were associated with less variation in SMA-negative villi villous barrier thickness, suggesting an association with placental maturation. These associations were independent of several maternal and toddler sociodemographic and perinatal characteristics. The associations of maternal depressive symptoms with placental morphology were the most consistent for depressive symptoms during the first pregnancy trimester, and they were also independent of maternal history of physician-diagnosed mental disorders before pregnancy. The associations of placental morphology with toddler psychiatric problems were also independent of maternal depressive symptoms concurrently to child assessment.

The villous barrier is the physical barrier through which maternal-fetal exchange of gases, nutrients and waste occurs. Its thickness directly correlates with exchange efficiency, thus it is a highly relevant physiological measurement. As gestation progresses the exchange demands placed on this barrier increase and the villous barrier of the SMA-negative villi becomes more heterogeneous in thickness. Fetal capillaries, especially their sinusoidal vessels in the terminal segments of the villous tree^23^, dilate into anuclear areas of the syncytium leading to areas of the villous barrier that are extremely thin to maximize exchange efficiency^24^. However, to accommodate these areas of thinning, syncytiotrophoblastic organelles accumulate in thicker areas outwith the VSMs, called syncytial knots^33^. Thus, an irregularity in villous barrier thickness develops and its appearance indicates optimal placental maturation. According to previous findings the development of VSMs and syncytial knots decrease diffusion resistance by 26%, compared with that of a barrier with uniform thickness^34^ and there is an inverse relationship between the number of VSMs and fetal hypoxia^26^. Analysis of uncomplicated full-term placentas showed that although absolute villous barrier thickness measurements varied between different placentas, the uniformity index (a ratio between the thick and thin villous barrier areas) was relatively constant from one placenta to another^34^. This indicates that there is likely an optimal irregularity of the villous barrier to maximally and optimally serve the growing fetus. Our data showing that less heterogeneity in placental villous barrier thickness is associated with maternal depressive symptoms could point to a mechanism whereby maternal depression-related changes cause subtle alterations in placental maturation, especially in the dynamic villous trophoblast layers. This may compromise the efficiency and robustness of materno-fetal exchange (diffusion controlled transport in thinned membrane areas and actively supported transport in thicker areas), which then affects fetal neurodevelopment, leading to psychiatric problems in toddlers. Among villous trophoblast layers, the most important for this finding could be the syncytiotrophoblast, which is the epithelium at the materno-fetal border and can be considered a steady state structure in a vulnerable sandwich position between cytotrophoblast proliferation and syncytial shedding^35^.

While maternal diabetes or hypertension in pregnancy did not explain the associations of placental morphology with maternal or toddler psychopathology, placentas from diabetic or hypertensive pregnancies had smaller vessel volumes in SMA-positive villi. The vessels within the SMA-positive villi are the fundamental conduits of blood supply from fetus to the SMA-negative peripheral part of the villous tree, which are the primary sites of materno-fetal exchange^15^. Any reduction in vessel volume within the more centrally located and larger SMA-positive villi would result in suboptimal blood supply to peripheral villi and hence compromised fetal nutrient and oxygen supply and waste removal^15^. Although the villous barrier thickness and hence the diffusing capacity of the barrier is unaltered in pregnancies with diabetic or hypertensive disorders^36,37^, if the carrying capacity of stem villi capillaries is compromised due to their scarceness, a deficient exchange may ensue due to blood supply from the fetus being limited by the suboptimal stem villi vessel volume.

The limitations of our study include the small sample size and use of only maternal reports of maternal depressive symptoms and toddler psychiatric problems. The sample size was 86 for the analyses on maternal depressive symptoms during pregnancy and placental morphology, and only 60 for the analyses on placental morphology and toddler psychiatric problems. Hence, there is a possibility for chance findings, and our findings need to be replicated in larger study samples.

Furthermore, attrition analyses suggested that some key characteristics of the study sample may be associated with study attrition, which may limit the generalizability of our findings. Since the women participating in the current study scored lower on antenatal depressive symptoms than the other women in the PREDO, and since birth register data suggests that none of the participating mothers had been diagnosed with mental disorders during pregnancy, the generalizability of the findings to more severe levels of maternal depression is questionable. However, we did detect associations with the same indicator of placental morphology for both continuously assessed antenatal depressive symptoms and for antenatal depressive symptoms exceeding a cutoff score for clinically relevant symptoms. Also the toddlers in the current study sample were younger than the other PREDO children at the time of completion of the CBCL, reflecting that sampling of placentas were added late to the PREDO study protocol, and some psychiatric problems assessed in the CBCL may not yet have developed to their full range of variation by 2–3 years of toddler age when psychiatric problems were assessed. Previous studies show that the level of internalizing problems increases between 1.5 and 5 years of age^3,38^. However, the age range of the current study sample is within the intended age range for the use of CBCL questionnaire (1.5–5 years), the scale has been validated for this whole age range^38–40^, and toddler age was not associated with toddler internalizing, externalizing, or total psychiatric problems in our study sample (r = 0.00, = −0.12, = −0.07, respectively, P-values ≥ 0.37).Yet, our findings need to be replicated also among older children.

Also, even though our explorative study indicated associations of the biological markers of placental morphology with maternal antenatal depressive symptoms and toddler psychiatric problems, we do not know which physiological factors underlie these associations. As stated, maternal depression during pregnancy is associated with physiological changes in the functioning of the neurobiological stress system, oxidative stress and maternal nutrition^6,9–14^. As such changes are also associated with placental morphology^41–45^ and/or with psychiatric problems in the offspring^46,47^, these factors may have contributed to the associations found. Furthermore, we cannot rule out or confirm genetic explanations for our findings, with some genetic vulnerabilities contributing both to maternal depressive symptoms, aberrations in placental development and increased psychiatric problems in toddlers. Further studies are needed to elucidate the contributory effects of genetic and environmental factors to our findings.

On the other hand, we circumvented the weakness of conventional histologic villous classifications (differentiation in stem, intermediate, and terminal villi) in the recognition of peripheral villous arborizations^48–50^ by detecting SMA as a criterion of more central (stem villus like, SMA-positive) or peripheral (SMA-negative) villi. However, care should be taken since many SMA-positive villi did not have a larger calibre than many SMA-negative villi, making them histologically indiscernible. The term stem villus is thus not a synonym of term SMA-positive villus. By 2D section histology, recognizing differences in arborization topology is impossible; nodes cannot be recognized. The recently introduced 3D microscopy of peripheral villous trees^48–50^ can potentially provide this information in further studies. Yet, our study has a novel focus, and its strengths include the longitudinal design, the use of validated questionnaires on maternal and toddler psychopathology^39,51–54^, and the repeated, fortnightly assessments of maternal antenatal depressive symptoms.

In summary, our novel, explorative, hypothesis-generating study showed that maternal depressive symptoms during pregnancy were associated with lower variability in the thickness of the villous membrane of the peripheral villi in term placentas. This lower variability, indicating a reduction in the variability in the villous barrier thickness of SMA-negative villi, was also associated with subsequent toddler psychiatric problems. We propose that this lower heterogeneity in villous barrier thickness may compromise materno-fetal exchange, suggesting a possible role for altered placental structure in the fetal programming of mental disorders^16^.

## Methods

Participants were from the prospective PREDO cohort^55^, which comprises 4777 pregnant women who gave birth to singleton live-born children in 2006–2010. These women were recruited to the study in early pregnancy at first ultrasound measurements at the antenatal clinics of ten study hospitals in Finland. All participating women signed informed consents. The PREDO study protocol was approved by Helsinki and Uusimaa Hospital District ethical committees^55^. The PREDO study has been conducted in accordance with the declaration of Helsinki.

Placental biopsy collection was introduced to the study protocol in 2009. Samples were collected at two maternity clinics in Helsinki, Finland. Placental morphology data were available from 96 mother-child dyads with infants born at term in 2009–2010. Eight had neither maternal nor child psychiatric data and were excluded from this study.

Our final study sample thus comprises 88 participants. Of them, 86 had data on maternal depressive symptoms during pregnancy and 60 on toddler psychiatric problems. Compared to other PREDO mothers, the mothers participating in this study had less depressive symptoms during the third pregnancy trimester (MD = −0.24 SD units: 95% CI = −0.45; −0.02: P = 0.03). Compared to non-participating PREDO children, the participating toddlers were younger at childhood follow-up (MD = −1.37 years: 95% CI = −1.30; −1.44: P < 0.001). There were no differences between participating and non-participating mothers or toddlers in other assessed characteristics (maternal age, education level, BMI, diabetes and hypertension in pregnancy, history of physician-diagnosed mental disorders before pregnancy, depressive symptoms at other time points, gestation length or toddler sex or psychiatric problems; P-values ≥ 0.08).

### Placental Morphology

Two placenta samples with an edge length of ~1 cm and full depth of the placenta were taken from each placenta after birth without a preference for specific placental sites from macroscopically unsuspicious areas, then routinely fixed in formaldehyde. After fixation lasting at least 24 hours, the samples were routinely dehydrated in an alcohol step gradient and embedded in paraffin. For morphometric analysis, these paraffin blocks were transferred to LMU Munich, Germany.

Paraffin samples were sectioned as 4–6 micrometres thick sections and placed on super frost object slides (SuperFrost plus, Thermofisher, Munich, Germany). All slides were deparaffinized through xylene and a stepwise ethanol gradient. Immunohistochemical double labelling was performed, comprising an antibody to CD34 (labelling of fetal endothelium) and to SMA (labelling of perivascular myofibroblast-like cells)^19^. Peroxidase with DAB as substrate (anti-CD34, brown reaction product) and β-galactosidase with X-Gal as substrate (anti-SMA, indigo-blue reaction product) were used for differential visualization in brightfield microscopy. Nuclei were counterstained with haematoxylin. The sequence of steps was empirically optimized such that the anti-CD34 detection was always carried out first and completely. Both immunohistochemical sequences used streptavidin-enzyme conjugates. Cross reactivity due to streptavidin use in both sequences was excluded by controls included in the second immunohistochemical sequence (anti-SMA). SMA-detection was used for classification of villi based on recommendations in the literature^49^. After peroxidase detection, anti-SMA detection was performed. Supplementary Tables S3–S4 give full details of the immunohistochemical reaction steps. Finally, the nuclei were counterstained with haematoxylin and the slides mounted with coverslips (Kaiser’s glycerol jelly, Merck, Darmstadt, Germany). All object slides were stored at 4 °C prior to microscopic evaluations.

**Figure 2:**
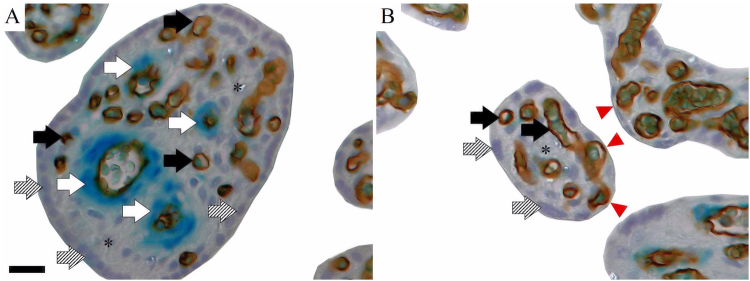
Immunohistochemical double labeling of perivascular sheath and fetal villous endothelium. (**A**) Shows an example of a γ-smooth-muscle-actin(SMA)-positive villus (indigo-blue in perivascular position; white arrows). The endothelium of fetal vessels is labelled (CD34: brown; black arrows), the villous stroma marked by asterisks and the trophoblast (bluish nuclei; shaded arrows) visible. (**B**) Exemplifies a SMA-negative villus (no SMA reactivity in perivascular position). The endothelium of fetal vessels is labelled (CD34: brown; black arrows), villous stroma marked by asterisks and trophoblast (bluish nuclei; shaded arrows) visible. Red arrow heads label vasculo-syncytial-membrane spots. (**A**) Scale bar is 25 µm and valid for (**B**).

**Figure 3:**
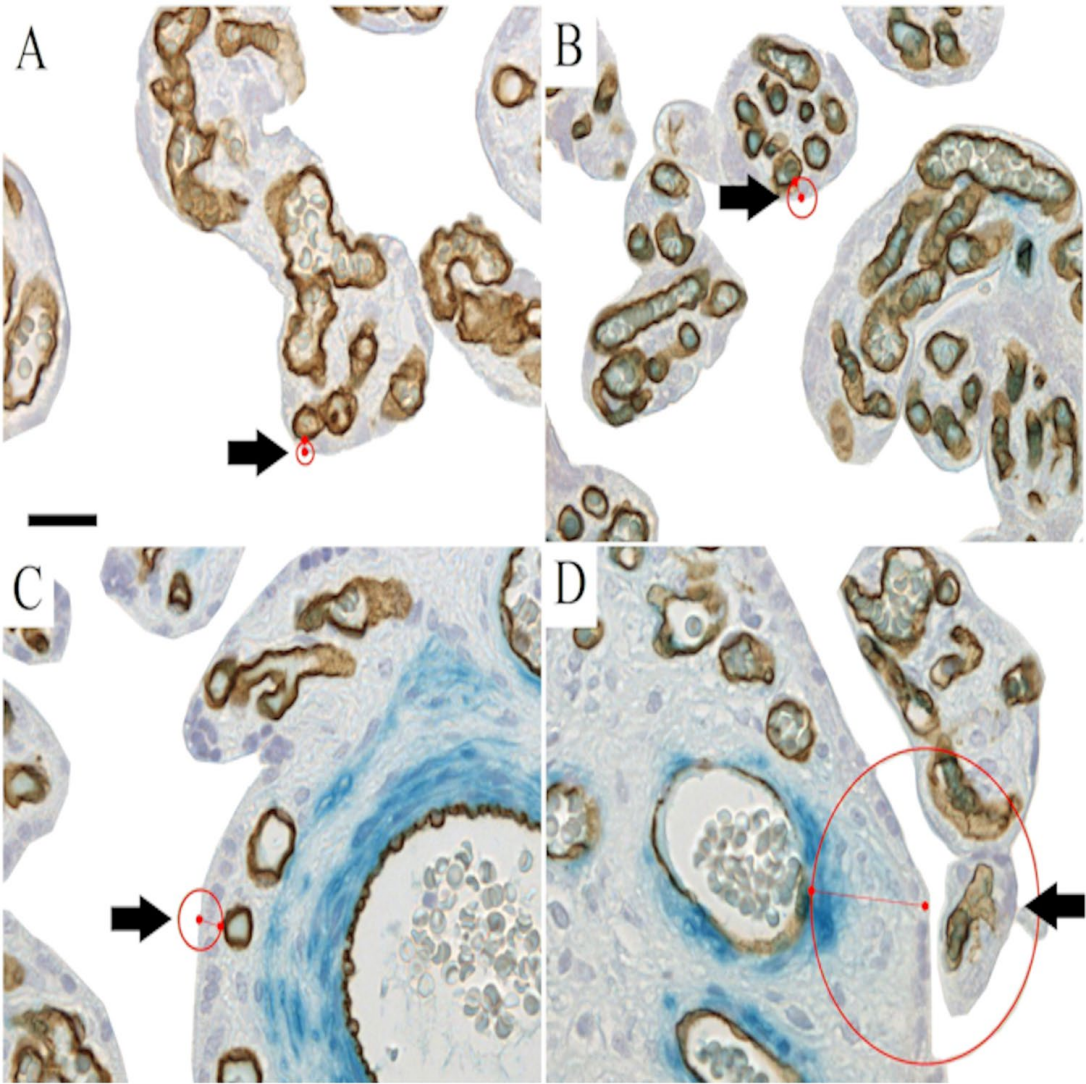
An illustration of the villous membrane thickness measurement principle. (**A**,**B**) Show examples of SMA-negative villi with endothelium labelled by CD34-reactivity (brown reaction product). (**C**,**D**) Show examples of SMA-positive villi (indigo-blue reaction product in perivascular position) with endothelium labelled by CD34-reactivity (brown reaction product). (**A**–**D**) We measured membrane thickness with the Nearest Neighbor analysis and the shortest distance (red line) from the trophoblast to the fetal endothelium by increasing a circle (red) from the trophoblast surface until first intersection with the endothelium. Exemplary measurement positions are marked with black arrows. Scale bar is 25 µm.

We examined nine placental morphology criteria: the volumes of SMA-positive and SMA-negative (peripheral) villi per placenta, the volume of capillaries per SMA-positive and SMA-negative villi per placenta, the ratio of volumes of SMA-positive to SMA-negative villi, the villous barrier thicknesses (distance from the outer trophoblast to the endothelium of the fetal vessels inside the villous tree) and the SDs of villous barrier thicknesses stratified by SMA-positive and SMA-negative villi. The volume criteria are expressed in millilitres and were calculated by dividing the assessed criterion by placental weight (grams). Volume estimates were performed according to the Cavalieri principle on single thin (4–6 micrometres) histological sections using a computerized stereology workstation, which comprised a modified light microscope (Axioskop;Zeiss) with motorized specimen stage for automatic sampling [MBF Bioscience, Williston, VT, United States of America (USA)] and stage controller [Type MAC 6000, Ludl Electronics, Hawthorne, New York, USA], focus encoder (Type MT 1271, Heidenhain, Germany), CCD colour video camera (1600H 3 1200 V pixels, MBF Bioscience, Williston, VT, USA) and stereology software (Stereo Investigator version 10;MBF Bioscience). This approach delivers volume fractions as raw data, which were stratified to the villous tree substructures (villous stroma, vessel lumen, endothelium, and syncytiotrophoblast), intervillous space and fibrinoid. Then, absolute volumes were calculated by multiplying volume fractions with total placental volume [placental volume is defined by placental weight divided by the density of placental tissue (1.03 g/millilitre)]. To determine villous barrier thickness, we used the Nearest Neighbor option in the stereology software (Stereo Investigator version 10, MBF Bioscience). The villous barrier thickness is expressed in micrometres. Figures 2 and 3 show illustrations of the placental morphology criteria measurements.

### Maternal Depressive Symptoms

The women assessed their depressive symptoms fortnightly up to 14 times during pregnancy from pregnancy weeks + days 12 + 0/13 + 6 until delivery or 38 + 0/39 + 6 pregnancy weeks + days with the Center for Epidemiological Studies Depression Scale (CES-D)^53^, which comprises 20 questions on the frequency of depressive symptoms during the preceding week. CES-D sum-scores range from 0 to 60. Higher scores indicate more depressive symptoms. The CES-D cutoff score of ≥16 indicates risk for clinical depression^53^. The CES-D has excellent psychometric properties^52–54,56,57^, and it has been validated also among pregnant women^57,58^. In our sample, the CES-D had high internal consistency (Cronbach’s α varying from =0.85 to =0.93).

### Toddler Psychiatric Problems

When the toddlers were 1.9–3.1 years old, the mothers completed the Child Behavior Checklist for Ages 1½-5 (CBCL1½-5) which is a well-validated and very widely used scale on child psychiatric problems^39,51^. It comprises 99 items, rated from 0 to 2. Higher t-scores reflect more problems on the CBCL1½-5 main scales of internalizing, externalizing and total problems^51^, on the seven CBCL1½-5 Syndrome Scales and on the five CBCL1½-5 Diagnostic and Statistical Manual for Mental Disorders-Fourth Edition-oriented scales^51^.

### Covariates

Information on maternal pre-pregnancy height and weight, of which pre-pregnancy BMI (kilograms/metres^2^) was calculated, maternal diabetes and/or hypertension in pregnancy (gestational and type 1 diabetes, preeclampsia, chronic and gestational hypertension; any vs. none), maternal age at delivery, toddler sex, birth date and gestation length was extracted from the Finnish Medical Birth Register and patient case records. Maternal history of physician-diagnosed mental disorders (depression, panic disorder, schizophrenia, other psychosis, other mental disorder) before pregnancy (no/yes; n = 80; 6 mothers with missing data were dummy-coded to an own category for regression analysis) and education level (primary/secondary vs. tertiary) was self-reported during pregnancy. Mothers assessed their depressive symptoms at toddler follow-up with the Beck Depression Inventory*-*II^59^. Toddler age at follow-up was calculated by subtracting birth date from CBCL1½-5 completion date.

### Statistical Analyses

To account for skewness and improve linear model fitting, the maternal depressive symptom scores, volumes of SMA-positive villi per placenta- and capillaries per SMA-positive villi per placenta, the villous barrier thickness of SMA-negative villi and the SD of villous barrier thickness of SMA-positive villi were square-root transformed and maternal pre-pregnancy body mass index and the placental ratio of the distribution of SMA-positive villi to SMA-negative villi, the villous barrier thickness of SMA-positive villi and the SD of villous barrier thickness of SMA-negative villi were rank-normalized according to Blom’s formula. All continuous variables are expressed in SD units (mean = 0: SD = 1).

We examined the associations between maternal depressive symptoms and placental morphology with linear regression models. In these models, trimester-weighted mean maternal depressive symptoms across pregnancy (mean value calculated from the value in first trimester and mean values across second and third trimesters) and mean scores during each pregnancy trimester and depressive symptoms above the clinical cutoff (≥16 across pregnancy or 0, 1, or 2/3 times across pregnancy trimesters) predicted placental morphology criteria. We present unstandardized regression coefficients (B) and 95% CI in SD units from unadjusted regression models, regression models adjusted for maternal age, education, BMI, diabetes and hypertension in pregnancy and gestation length, and models adjusted further for maternal history of physician-diagnosed mental disorders before pregnancy.

We tested whether placental morphology criteria predicted toddler internalizing, externalizing and total problems using linear regression. We present B and 95% CI from unadjusted models, models adjusted for toddler age and sex, gestation length, maternal age, education, BMI, diabetes and hypertension in pregnancy, and models adjusted for maternal depressive symptoms concurrent to rating toddler’s psychiatric problems. To specify any possible effects of toddler psychiatric problems, we examined the syndrome- and DSM-IV-oriented CBCL as secondary outcomes for those placental criterion that showed significant associations with the main scales. The associations with these CBCL subscales were examined with tobit regressions, since these scales are left-censored^51^.

### Data Availability

The datasets generated during and/or analysed during the current study are not publicly available due to prohibitions by national laws since the data include patient report data. However, the datasets generated during and/or analysed during the current study are available from the corresponding author on reasonable request in completely anonymized form. Due to the sensitive nature of the patient report data, data requests may require further approval by the PREDO Study Board, that also enables collaboration in PREDO data analysis through specific study proposals^55^.

## Electronic supplementary material

Supplementary Tables
