# Supplementary material for: Placental Morphology Is Associated with Maternal Depressive Symptoms during Pregnancy and Toddler Psychiatric Problems
Source: Sci Rep. 2018 Jan 15;8:791. doi: 10.1038/s41598-017-19133-9 (PMC5768752; doi:10.1038/s41598-017-19133-9)
Supplement: Supplementary file 1 — Supplementary Tables [file 41598_2017_19133_MOESM1_ESM.pdf]

Placental Morphology Is Associated with Maternal Depressive Symptoms during Pregnancy and Toddler Psychiatric Problems

Marius Lahti-Pulkkinen*, Melissa Jane Cudmore*, Eva Haeussner, Christoph Schmitz, Anu-Katriina Pesonen, Esa Hämäläinen, Pia M Villa, Susanna Mehtälä, Eero Kajantie, Hannele Laivuori, Rebecca M Reynolds, Hans-Georg Frank, Katri Räikkönen

*Joined first authorship

Supplementary Table S1. The associations between the different placental morphology indicators. Pearson correlation coefficients and p-values.

	Volume of capillaries per SMA-positive villi per placenta		Volume of SMA-negative villi per placenta		Volume of capillaries per SMA-negative villi per placenta		Ratio of the distribution of stem villi to SMA-negative villi		Villous barrier thickness of SMA-positive villi		Standard deviation of villous barrier thickness of SMA-positive villi		Villous barrier thickness of SMA-negative villi		Standard deviation of villous barrier thickness of SMA-negative villi	
	r	p	r	p	r	p	r	p	r	p	r	p	r	p	r	p
Volume of SMA-positive villi per placenta	0.92	<0.001	-0.16	0.13	-0.11	0.29	0.87	<0.001	-0.08	0.44	0.18	0.10	-0.09	0.43	-0.18	0.10
Volume of capillaries per SMA-positive villi per placenta			-0.23	0.03	-0.17	0.10	0.84	<0.001	-0.19	0.08	0.10	0.38	-0.04	0.74	-0.13	0.24
Volume of SMA-negative villi per placenta					0.96	<0.001	-0.59	<0.001	0.20	0.06	0.09	0.40	0.04	0.69	0.12	0.27
Volume of capillaries per SMA-negative villi per placenta							-0.54	<0.001	0.20	0.06	0.10	0.36	0.04	0.73	0.15	0.18
Ratio of the distribution of stem villi to SMA-negative villi									-0.16	0.14	0.12	0.29	-0.08	0.46	-0.19	0.07
Villous barrier thickness of SMA-positive villi											0.62	<0.001	0.32	0.002	0.23	0.03
Standard deviation of villous barrier thickness of SMA-positive villi													0.15	0.17	0.15	0.15
Villous barrier thickness of SMA-negative villi															0.72	<0.001

Supplementary Table S2. Standard deviation of villous barrier thickness of SMA-negative villi and Toddler Psychiatric Problems (n=60). Child Behavior Checklist Syndrome and Diagnostic and Statistical Manual for Mental Disorders, Fourth Edition-Oriented Scales.

**Child Behavior Checklist Toddler Psychiatric Problem Scales**	**Standard deviation of villous barrier thickness of SMA-negative villi**					
	**Model 1**		**Model 2**		**Model 3**	
**Syndrome Scales**	**B(95% CI)**	**p**	**B(95% CI)**	**p**	**B(95% CI)**	**p**
Emotionally Reactive Problems	-0.76(-1.40;-0.12)	0.02	-0.64(-1.21;0.06)	0.03	-0.69(-1.31;-0.07)	0.03
Anxious/Depressed Problems	-0.90(-1.97;0.17)	0.10	-1.18(-2.14;-0.21)	0.02	-0.78(-1.83;0.27)	0.14
Somatic Complaints	-0.40(-0.92;0.13)	0.14	-0.31(-0.82;0.19)	0.23	-0.36(-0.88;0.16)	0.18
Withdrawn Problems	-0.36(-0.80;0.07)	0.10	-0.42(-0.85;0.01)	0.06	-0.31(-0.72;0.11)	0.15
Sleep Problems	-0.99(-1.59;-0.40)	0.001	-1.01(-1.60;-0.43)	0.002	-0.90(-1.47;-0.33)	0.002
Attention Problems	-0.10(-0.62;0.43)	0.72	-0.11(-0.61;0.40)	0.69	-0.02(-0.53;0.49)	0.93
Aggressive Behaviour Problems	-0.47(-1.04;0.10)	0.11	-0.47(-1.03;0.09)	0.10	-0.38(-0.92;0.16)	0.17
**Diagnostic and Statistical Manual for Mental Disorders,**						
Affective Problems	-0.55(-0.90;-0.20)	0.002	-0.57(-0.92;-0.22)	0.002	-0.52(-0.88;-0.17)	0.004
Anxiety Problems	-1.11(-1.90;-0.32)	0.01	-1.24(-1.99;-0.48)	0.001	-1.00(-1.75;-0.24)	0.01
Pervasive Developmental Problems	-0.38(-0.76;-0.00)	0.05	-0.38(-0.75;-0.01)	0.05	-0.33(-0.70;0.03)	0.08
ADHD Problems	-0.30(-0.95;0.36)	0.37	-0.33(-0.97;-0.31)	0.32	-0.23(-0.87;0.41)	0.49
Oppositional Defiant Problems	-0.40(-0.84;0.04)	0.08	-0.43(-0.85;-0.01)	0.05	-0.33(-0.75;0.09)	0.12

SMA= γ-smooth muscle actin.
Unstandardized regression coefficients (B) and their 95 % Confidence Intervals (CI) from Tobit Regression Models. Both the predictor and outcome variables are expressed in standard deviation units.
Model 1 is unadjusted.
Model 2 is adjusted for child's age and sex, gestation length, maternal age at childbirth, education level, pre-pregnancy body mass index and maternal hypertensive and diabetic disorders in pregnancy.
Model 3 is adjusted for maternal depressive symptoms concurrently to assessing the child.

Supplementary Table S3. The full protocol of the first immunohistochemical sequence.			
**Step**	**Processing**	**Solution**	**Time**
1	Blocking of the endogenous peroxidase	3% H_2O_2 (Merck, Darmstadt, Germany) in PBS buffer	30 min
2	Washing	Phosphate-buffered saline (PBS)	5 min
3	Blocking	5 % goat serum (GOS; Vector Labs, Burlingame, USA) in PBS	30 min
4	Incubation of the first antibody anti-CD34 (Thermo Fisher Scientific, Fremont, USA)	1:900 solution: 5 % GOS-PBS + CD34	45 min
5	Washing	PBS	3 x 5 min
6	Incubation with the second biotinylated goat-anti-mouse-IgM-antibody (Vector Labs, Burlingame, USA)	1:200 solution: 2% Bovine serum albumin (BSA; Sigma-Aldrich, Munich, Germany) in PBS + second antibody	45 min
7	Washing	PBS	3 x 5 min
8	Incubation with Avidin-peroxidase-conjugate (Vector Labs, Burlingame, USA)	PBS + Avidin + biotinylated horseradish-peroxidase	45 min
9	Washing	PBS	3 x 5 min
10	peroxidase-detection with diaminobenzidin (DAB, Sigma-Aldrich, Munich, Germany)	DAB + H_2O_2	20 min
11	Washing	tap water	shortly
12	Washing	PBS	2 x 5 min

**Supplementary Table S4. The full protocol of the second immunohistochemical sequence.**			
**Step**	**Processing**	**Solution**	**Time**
1	Washing	PBS	5 min
2	Blocking	5% GOS (Sigma-Aldrich, Munich, Germany) in PBS	30 min
3	Incubation with the first antibody γ-sm-Actin (MP Biomedicals, Eschwege, Germany)	1:900 solution: 1% BSA-PBS buffer + γ-sm-Actin	120 min
4	Washing	PBS	3 x 5 min
5	Incubation with the second biotinylated goat-anti-mouse-IgM-antibody (Vector Labs, Burtingame, USA)	1:200 solution: 5% GOS-PBS + second antibody	45 min
6	Washing	PBS	3 x 5 min
7	Incubation of streptavidin-β-galactosidase-conjugate (Life Technologies, Darmstadt, Germany).	1:100 solution: PBS+ streptavidin-β-galactosidase-conjugate	40 min
8	Washing	PBS	3 x 5 min
9	Detection of galactosidase with X-Gal (Invitrogen, Carlsbad, CA, USA)	1:40 solution: X-Gal buffer + X-Gal stock solution<br>X-Gal buffer: Potassium Ferricyanide Crystalline (Merck, Darmstad, Germany), Potassium Ferricyanide Trihydrate (Sigma-Aldrich, Munich, Germany), Magnesiumchlorid (Sigma-Aldrich, Munich, Germany), PBS<br>X-Gal stock solution: X-Gal in 4% Dimethylformamide (DMF; Merck, Darmstadt, Germany)	25 min
10	Washing	PBS	3 x 5 min
11	Washing	Distilled water	shortly
12	Counterstaining	Hematoxylin	5 sec
13	Bluing	Tap water	5min
14	Washing	Distilled water	shortly
